# Stable expression of mucin glycoproteins GP40 and GP15 of* Cryptosporidium parvum* in *Toxoplasma gondii*

**DOI:** 10.1186/s13071-024-06159-y

**Published:** 2024-02-15

**Authors:** Muxiao Li, Xiaohua Sun, Haoyu Chen, Na Li, Yaoyu Feng, Lihua Xiao, Yaqiong Guo

**Affiliations:** State Key Laboratory for Animal Disease Control and Prevention, South China Agricultural University, Guangzhou, 510642 China

**Keywords:** *Cryptosporidium parvum*, *Toxoplasma gondii*, GP40, Glycoprotein, Expression

## Abstract

**Background:**

*Cryptosporidium* spp. are common protozoa causing diarrhea in humans and animals. There are currently only one FDA-approved drug and no vaccines for cryptosporidiosis, largely due to the limited knowledge of the molecular mechanisms involved in the invasion of the pathogens. Previous studies have shown that GP60, which is cleaved into GP40 and GP15 after expression, is an immunodominant mucin protein involved in the invasion of *Cryptosporidium*. The protein is highly O-glycosylated, and recombinant proteins expressed in prokaryotic systems are non-functional. Therefore, few studies have investigated the function of GP40 and GP15.

**Methods:**

To obtain recombinant GP40 with correct post-translational modifications, we used CRISPR/Cas9 technology to insert *GP40* and *GP15* into the *UPRT* locus of *Toxoplasma gondii*, allowing heterologous expression of *Cryptosporidium* proteins. In addition, the Twin-Strep tag was inserted after *GP40* for efficient purification of GP40.

**Results:**

Western blotting and immunofluorescent microscopic analyses both indicated that GP40 and GP15 were stably expressed in *T. gondii* mutants. GP40 localized not only in the cytoplasm of tachyzoites but also in the parasitophorous vacuoles, while GP15 without the GPI anchor was expressed only in the cytoplasm. In addition, a large amount of rec*Tg*GP40 was purified using Strep-TactinXT supported by a visible band of ~ 50 kDa in SDS-PAGE.

**Conclusions:**

The establishment of a robust and efficient heterologous expression system of GP40 in *T. gondii* represents a novel approach and concept for investigating *Cryptosporidium* mucins, overcoming the limitations of previous studies that relied on unstable transient transfection, which involved complex steps and high costs.

**Graphical Abstract:**

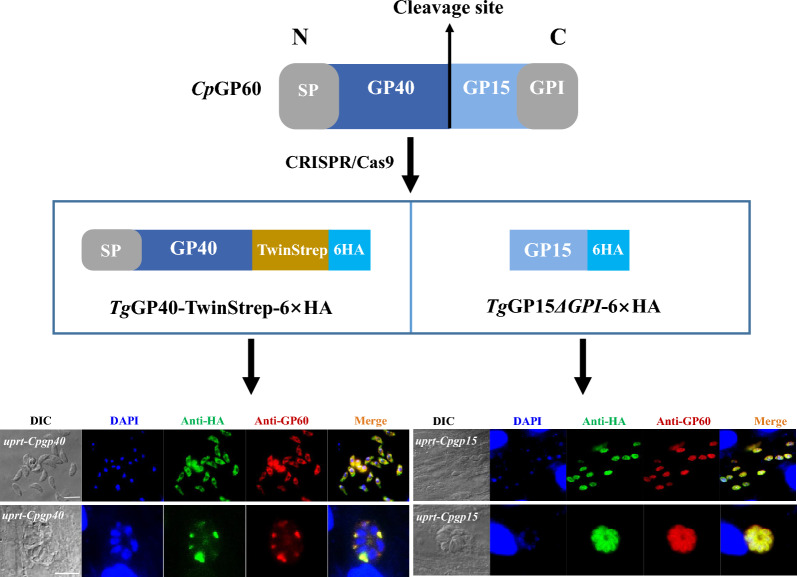

**Supplementary Information:**

The online version contains supplementary material available at 10.1186/s13071-024-06159-y.

## Background

*Cryptosporidium* spp. primarily infect the intestinal epithelial cells of humans and a variety of animals, causing diarrhea and associated disease [1]. They are transmitted through the fecal-oral route [2], and they cause cryptosporidiosis, which is the second leading cause of childhood diarrhea in middle and low-income countries and a major cause of mortality in individuals with HIV/AIDS [3]. Nitazoxanide is the only FDA-approved drug against human cryptosporidiosis, and there is no available vaccine against this infection [4].

An established strategy for the identification of potential drug targets or vaccine candidates in *Cryptosporidium* involves the exploration of surface antigens that participate in the recognition, attachment, and invasion of host epithelial cells, as well as the investigation of their interactions with the host [5]. A family of mucin glycoproteins is involved in the invasion of *Cryptosporidium* into host cells [6]. These mucins undergo significant O-linked glycosylation modifications, which have been shown to be crucial for their proper folding and function [7]. Among them, GP60 emerges as one of the most prominent mucin proteins in the invasion of *Cryptosporidium* [8]. This protein is cleaved into GP40 and GP15 after its expression, and GP40 remains on the parasite surface by binding to GP15, which has a GPI anchor [9, 10]. GP40 has > 30 O-linked glycosylation sites, while GP15 has a few such sites. In 2000, several research groups reported the discovery of the gene encoding GP60. However, few studies have investigated its function. This is mainly due to the challenges associated with conducting in-depth studies of mucins using recombinant proteins expressed in *Escherichia coli*, which have no biological function [9, 11–13].

Heterologous expression of *Cryptosporidium* mucins in *Toxoplasma gondii* probably provides recombinant proteins with the correct post-translational modifications. *Toxoplasma gondii* and *Cryptosporidium* belong to the phylum Apicomplexa, and *T. gondii* is amenable to in vitro cultivation [14]. In addition, well-established genetic manipulation techniques such as CRISPR/Cas9 have been applied to *T. gondii* [15]. In 2003, the transient expression of recombinant GP40/15 was achieved using the RH*Δhxgprt* strain of *T. gondii*, and mass spectrometry analysis confirmed that the recombinant GP40/15 had the same O-linked glycosylation as the native GP40/15 [16]. The researchers further improved the stability of the system by modifying the expression plasmid, enabling the successful affinity purification of recombinant GP40 [17]. These suggest that the heterologous expression system of *T. gondii* can be utilized for the expression and production of *Cryptosporidium* mucins.

Despite the incorporation of the episomal shuttle sequence to mitigate plasmid loss associated with transient expression of foreign proteins in *T. gondii*, the plasmid remains susceptible to loss during the passage, freezing, and recovery of parasites [18]. In addition, the high cost of HA-tag affinity purification poses a challenge to the production of recombinant proteins for biological studies. In view of these limitations, we employed CRISPR/Cas9 technology to insert *GP40* and *GP15* into the *UPRT* locus of *T. gondii,* respectively [15]. In addition, the Twin-Strep tag was used for protein purification, which offers improved purification efficiency at a more affordable cost [19–21]. As a result, a more robust and efficient heterologous expression system of *T. gondii* was established, providing a novel approach and concept for the study of *Cryptosporidium* mucins.

## Methods

### Parasite strains

The IIdA20G1-HLJ isolate of *Cryptosporidium parvum* was originated from a dairy calf in Heilongjiang (HLJ) Province, China, in 2018 [22]. The oocysts of IIdA20G1-HLJ were initially recovered from a fecal sample from the calf by gradient centrifugation, maintained by passage in IFN-γ knockout (GKO) mice, and stored at 4 °C in PBS containing antibiotics for < 3 months prior to use in the study [23, 24]. The RH*Δku80* strain of *T. gondii* was obtained from Dr. Bang Shen at Huazhong Agricultural University in 2021. It was continuously passaged in human foreskin fibroblast (HFF, ATCC SCRC-1041, Manassas, VA, USA) monolayers and frozen in liquid nitrogen for long-time storge. Prior to use in the study, the RH*Δku80* strain was recovered from the liquid nitrogen and passaged in HFF monolayers. After 2–3 days of culture, the fresh tachyzoites of the RH*Δku80* strain in the HFF culture were harvested by differential centrifugation.

### HFF cell culture and infection of *T. gondii*

The HFF cells were used for in vitro cultivation of *T. gondii*. The cells were seeded in flasks (T25 and T175) or plates (24-well and 96-well) and cultured in DMEM high-glucose medium (Thermo, Waltham, MA, USA) supplemented with 10% fetal bovine serum (FBS; Thermo), 1% l-glutamine (Sigma Aldrich, St. Louis, MO, USA), and 1% antibiotic antimycotic solution (Sigma Aldrich) at 37 °C in a 5% CO_2_ atmosphere until they reached approximately 80% confluence. Tachyzoites of the RH*Δku80* strain or the mutants generated in the study were allowed to infect cells in confluent cultures in DMEM high-glucose medium supplemented with 2% FBS [25].

### Plasmid construction

A specific CRISPR/Cas9 plasmid (p*SAG1*-Cas9-*TgU6*-sg*TgUPRT*) was obtained from Dr. Bang Shen, Huazhong Agricultural University, in 2021 [15]. Homology repair template plasmids capable of overexpressing *Cp*GP40 or *Cp*GP15 were generated by Gibson assembly cloning using the ClonExpress MultiS One Step Cloning Kit (Vazyme, Nanjing, China) according to the procedure. To construct repair plasmids, *GP40* (633 bp) and *GP15Δgpi* (228 bp) were first amplified from the genomic DNA extracted from the *C. parvum* IIdA20G1-HLJ isolate. Subsequently, a portion of the *UPRT* 5′-homologous (1178 bp), 3′-homologous (994 bp), *TgGRA1* promoter (451 bp), and *TgGRA2* 3′UTR (398 bp), were amplified from the genomic DNA of *T*. *gondii*, respectively. The DHFR*-TS (3513 bp) and *pUC19* (2769 bp) backbone was amplified from the *pUC19*-loxp-DHFR*-amp plasmid provided by Dr. Bang Shen in 2021. After the plasmid *TgUPRT* 5′UTR-CpGP40-DHFR*-TgUPRT 3′UTR was completed by Gibson assembly cloning with the sequences amplified, the Twin-Strep and 6 × HA tags were inserted into the C-terminus of GP40 or GP15 using the same approach to finally generate the plasmid *TgUPRT* 5′UTR-*CpGP40*-Twinstrep-6 × HA-DHFR*-TgUPRT 3′UTR or *TgUPRT* 5′UTR-*CpGP15*-6 × HA-DHFR*-*TgUPRT* 3′UTR. Subsequently, these homology repair plasmids were then used as templates in PCR amplifications to obtain enough linearized homologous templates for transfection. The primers used (p*GRA1*-*Cpgene*-*TerGRA2*-DHFR*-F/R) are listed in Additional file 1: Table S1.

### Generation of the RH*Δku80 Tguprt*-*Cpgp40* and* Tguprt*-*Cpgp15Δgpi* mutants

**Fig. 1:**
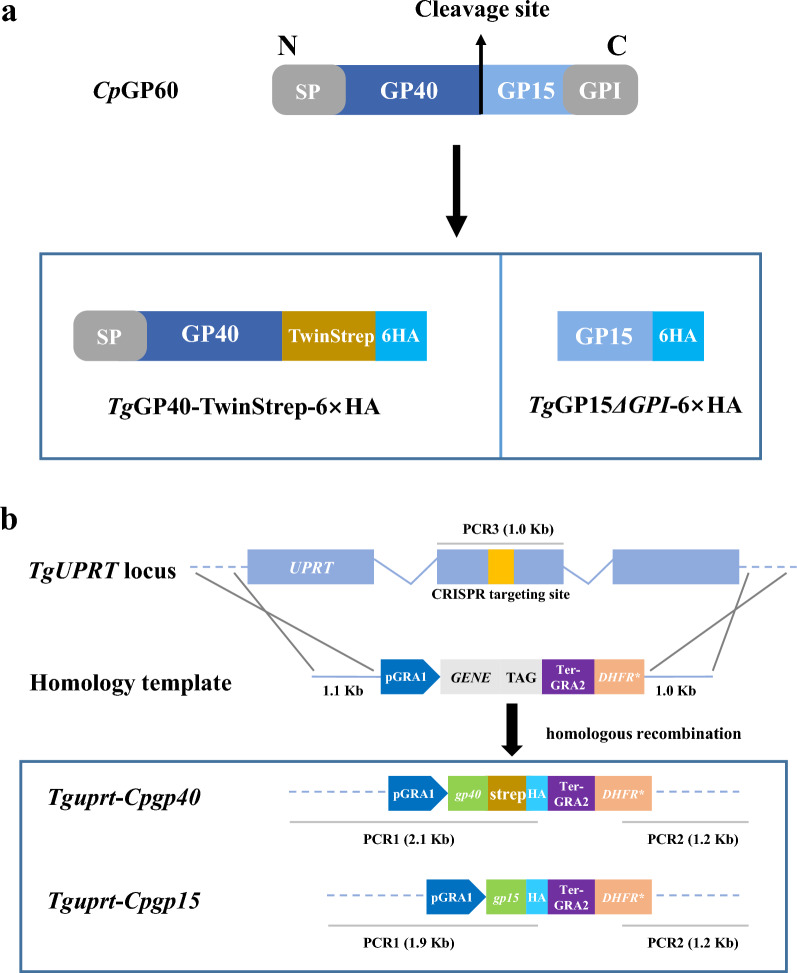
Strategy used in the heterologous expression of GP40 and GP15 of *Cryptosporidium parvum* in *Toxoplasma gondii*. **a** The *C. parvum* GP60 is composed of GP40 and GP15, with a signal peptide at the N-terminus, a GPI anchor at the C-terminus, and a furin cleavage site RSRR between the two cleavage products. In this study, GP40 was truncated and fused to the Twinstrep-6 × HA tag for expression, retaining the signal peptide of GP40 while removing the furin cleavage site RSRR. GP15 without GPI was fused to the 6 × HA tag for expression. **b** Schematic illustration of the CRISPR/Cas9 strategy used to generate RH*Δku80* mutant (*Tguprt*-*Cpgp40* and *Tguprt*-*Cpgp15Δgpi*) by inserting *CpGP40*-Twinstrep-6 × HA-DHFR* (pyrimethamine-resistant DHFR) or *CpGP15Δgpi*-6 × HA-DHFR*. The transfection of the sg*UPRT* together with an amplicon containing a *CpGP40*-Twinstrep-6 × HA-DHFR* (*CpGP15Δgpi*-6 × HA-DHFR*)-expressing cassette flanked by homology regions to *UPRT* was used to generate the gene insertion. The orange bar in *UPRT* gene represents the region targeted by the sgRNA

Fresh tachyzoites (2 × 10^7^ per transfection) of the harvested RH*Δku80* strain were resuspended in 250 μl transfection buffer cytomix; 7.5 μg CRISPR/Cas9 plasmids and 1.5 μg linearized homology repair templates were added to the mixture containing tachyzoites and cytomix, which was then transferred to a cuvette (BioRad, Hercules, CA, USA) and electroporated twice (1600 V, 25 μF, 50 Ω) using a BTX Gemini system (BTX, Holliston, MA, USA). The transfected tachyzoites were allowed to infect HFF cells. After about 18 h of culture, 1 μM pyrimethamine (Life Technologies, Carlsbad, CA, USA) and 10 μM floxuridine (Thermo) were added to the culture medium for selection of the mutants. After 2–3 passages, 0–1 of tachyzoite obtained by limiting dilution was allowed to infect cells cultured in 96-well plates to obtained single colony. PCRs targeting three different sites of each mutant were used to confirm the correct integration of templates as previously described [26]. Briefly, DNA was extracted from the culture of each mutant using the TIANamp Blood DNA Midi Kit (Tiagen Biotech, Beijing, China), and used as the template in the PCR analyses. The three sites of each mutant amplified by PCR are shown in Fig. 1b, and the primers used are listed in Additional file 1: Table S1.

### Immunofluorescence verification of rec*Tg*GP40 and rec*Tg*GP15

The expression of *C. parvum* GP40 and GP15*Δgpi* in *T. gondii* was initially verified by immunofluorescence assay (IFA). For the IFA analysis of the RH*Δku80 Tguprt*-*Cpgp40* mutant and the *Tguprt*-*Cpgp15Δgpi* mutant, HFF cells were infected with 10^5^ tachyzoites of these two mutants, respectively. After 16 h of culture at 37 °C with 5% CO_2_, the HFF cells were fixed with 4% paraformaldehyde for 15 min, permeabilized with 0.1% Triton X-100 at 37 °C for 10 min, and blocked with 1% BSA at 37 °C for 20 min. The following primary antibodies were diluted in 0.1% BSA for staining: mouse anti-HA (MBL Beijing Biotech, Beijing, China) at a 1:500 dilution, mouse anti-GP60 (purified rabbit pAbs) at 1:800 dilution. Cells were incubated with the antibodies for 40 min at 37 °C, followed by six washes with PBS. Alexa Fluor-conjugated secondary antibodies (Thermo) diluted 1:1000 and Hochest diluted 1:2000 in 0.1% BSA were added to the coverslips and incubated at 37 °C for 20 min. The cells were then washed six times with PBS and mounted on slides using Vectashield. The stained slides were examined using a Zeiss Axioskop Mot 2 fluorescence microscope (Carl Zeiss, Oberkochen, Germany). Images were manipulated using ZEN microscopy software (Carl Zeiss, Oberkochen, Germany).

### Western blot analysis of rec*Tg*GP40 and rec*Tg*GP15

The expression of *C. parvum* GP40 in *T. gondii* was further verified by Western blot analysis using rabbit anti-GP60 (purified rabbit pAbs). The RH*Δku80 Tguprt*-*Cpgp40* mutant (10^5^ tachyzoites) was grown in HFF cells cultured in DMEM medium for 24 h. All parasites were then purified and collected by differential centrifugation, and total proteins of the parasites were extracted using RIPA lysis buffer (Thermo) by incubation at 4 °C. The lysis was centrifugated, and 50 μl of the supernatant was used for Western blot analyses. The PVDF membrane was blocked in TBST (0.05% Tween 20 in TBS) containing 1% BSA and then incubated with the primary antibodies. The following primary antibodies were used and diluted in TBST containing 0.1% BSA: mouse anti-HA (MBL Beijing Biotech, Beijing, China) at 1:1000 dilution; rabbit anti-GP60 (purified rabbit pAbs) at 1:800 dilution. After thoroughly washing with TBST, the membrane was then incubated with HRP-conjugated antibodies (Beyotime, Shanghai, China) diluted 1:3000 in TBST containing 0.1% BSA. After washing with TBST, the membrane was incubated with Immobilon Western Chemiluminescent HRP Substrate (Millipore, Massachusetts, USA) and analyzed using a Chemstudio imaging system (Analytik Jena, Jena, Germany).

### Purification of rec*Tg*GP40

Strep-TactinXT resin (IBA GmbH, Göttingen, Germany) was used to purify rec*Tg*GP40-Twinstrep-HA as shown in Fig. 4a. In this procedure, the RH*Δku80 Tguprt*-*Cpgp40* mutant was grown in HFF cells cultured in DMEM medium for 24 h. The cultures were then lysed with RIPA lysis buffer and centrifuged at 16,000*g* and 4 °C for 10 min to obtain the supernatant. The Strep-Tactin column with resin was equilibrated with wash buffer (100 mM Tris/HCl pH 8.0, 150 mM NaCl, 1 mM EDTA) and then combined with the supernatant. After the supernatant had completely entered the column, the column was successively washed with wash buffer and elution buffer (wash buffer containing 2.5 mM desthiobiotin). The eluate was further concentrated by dialysis, and 100 μl of the concentrated eluate was used for identification of protein bands by SDS-PAGE, Western blot, and liquid chromatography/mass spectrometry (LC/MS).

## Results

### Construction of RH*Δku80 Tguprt*-*Cpgp40* mutant and *Tguprt*-*Cpgp15Δgpi* mutant

**Fig. 2:**
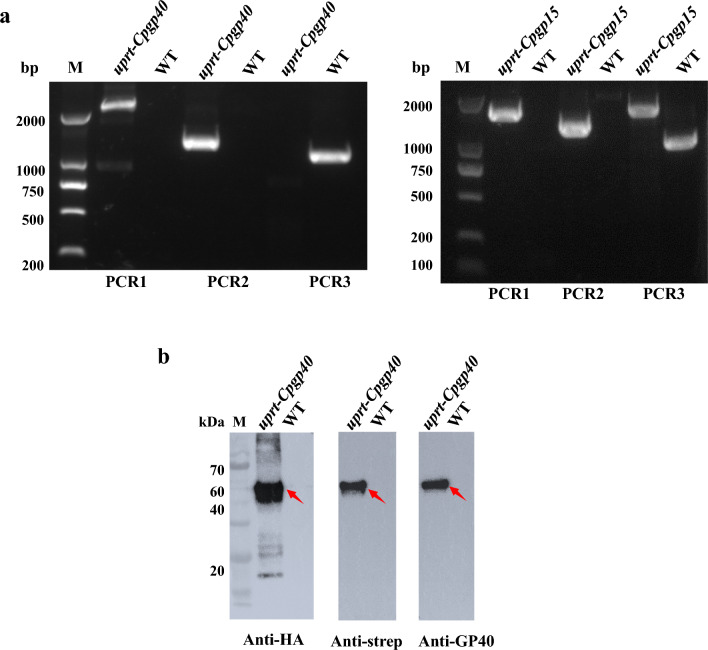
Verification of the correct integration and expression of the GP40 and GP15*ΔGPI* of *Cryptosporidium parvum* in *Toxoplasma gondii*. **a** Diagnostic PCR confirming the homologous integration and gene disruption in a representative clone compared to the parental line RH*Δku80*. PCR1 and PCR2 provide evidence of homologous integration based on products amplified between the *CpGP40*-*Twinstrep*-6 × HA-DHFR* (or *CpGP15*-6 × HA-DHFR*) gene and regions in the *UPRT* locus that lie outside of the targeting amplicon. PCR3 amplifies a 1.0-kb fragment in wild-type (WT) parasites that is lost because of the insertion of *CpGP40*-*Twinstrep*-6 × HA-DHFR* (or *CpGP15Δgpi*-6 × HA-DHFR*). **b** Expression of *Cp*GP40-Twinstrep-6 × HA in *Tguprt*-*Cpgp40* mutant determined by Western blotting. *Cp*GP40 was detected with mouse anti-HA, rabbit anti-Strep, and rabbit anti-GP60, with the WT control

For the expression of *Cp*GP40 and *Cp*GP15 in *T. gondii*, we attempted to insert the *GP40* and *GP15* into the *UPRT* locus of the RH*Δku80* strain using CRISPR/Cas9 (Fig. 1b). To facilitate the purification of the recombinant protein, GP40 was truncated by removing the RSRR site, and a TwinStrep-6 × HA tag was added to the C-terminus of GP40 (Fig. 1). The HA tag was used for the identification of the mutants, and the Twin-Strep tag was used for purification of the recombinant proteins. The RH*Δku80 Tguprt*-*Cpgp40* mutant and the RH*Δku80 Tguprt*-*Cpgp15* mutant were obtained after selection of pyrimethamine and floxuridine, and limiting dilution. In PCR analyses of the three sites of the mutants, expected bands were present in the PCR1 and PCR2 analyses of the RH*Δku80 Tguprt*-*Cpgp40* mutant and RH*Δku80 Tguprt*-*Cpgp15* mutant, while no band was observed in the PCR1 and PCR2 analyses of the RH*Δku80* strain (WT). This confirmed that *CpGP40* and *CpGP15* were correctly inserted into the *UPRT* locus. In the PCR3 analysis, the sequence covering the CRISPR targeting site (the *UPRT* locus) was amplified in the RH*Δku80* strain (WT). In contrast, no bands were present in the PCR3 analysis of the RH*Δku80 Tguprt*-*Cpgp40* mutant and the RH*Δku80 Tguprt*-*Cpgp15* mutant, which confirmed that the *UPRT* was knockout in the two mutants (Fig. 2a).

### Expression of GP40 in fusion with the Twin-Strep tag and HA tag

**Fig. 3:**
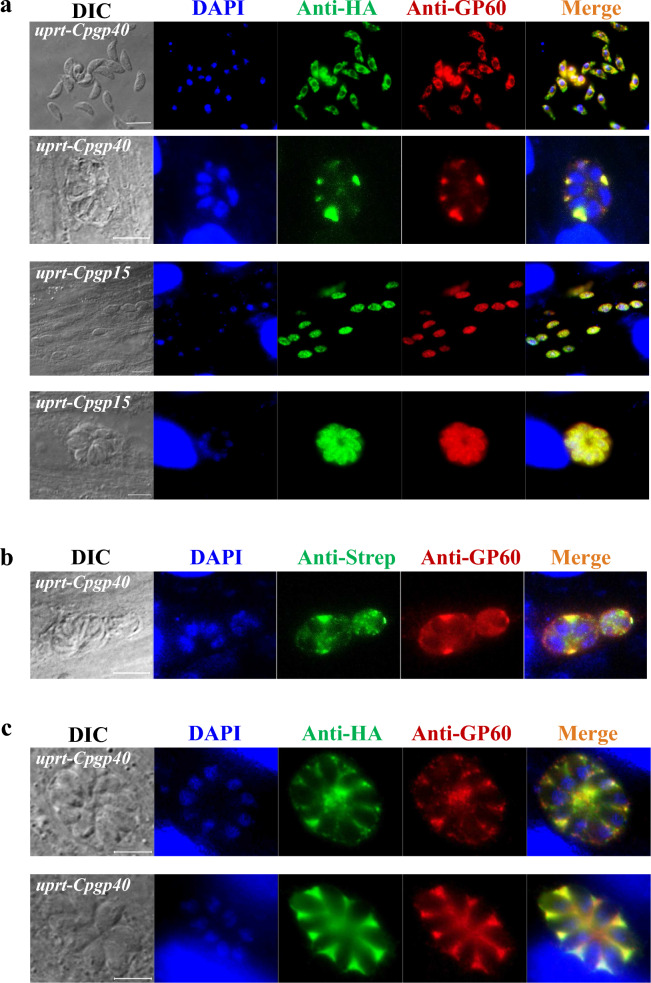
Verification of the expression of the GP40 and GP15*ΔGPI* of *Cryptosporidium parvum* in *Toxoplasma gondii* by immunofluorescence assay. **a** *Cp*GP40 and *Cp*GP15*ΔGPI* expressed in *T. gondii*. *T. gondii* RH*Δku80* tachyzoites were transfected with p*GRA1*-*CpGP40* or p*GRA1*-*CpGP15Δgpi* and allowed to infect HFF monolayers. IFA was performed 24 h after infection with anti-GP60 and -HA followed by Alexa Fluor-488-conjugated and 594-conjugated secondary antibodies. The far left panel shows the DIC field, and the far right panel shows a merged fluorescence image of the same field. DAPI-stained nuclei are in blue, Alexa Fluor-488 is in green, and Alexa Fluor-594 is in red. **b** *Cp*GP40 expressed in *T. gondii*. IFA was performed 24 h after infection with anti-GP60 and -Strep followed by Alexa Fluor-488-conjugated and 594-conjugated secondary antibodies. **c** High-power images of the IFA to show the fine localization of the GP40 expression. *Cp*GP40 proteins expressed in *T. gondii* are localized to the parasitophorous vacuole. Scale bars = 5 μm

GP40 was regulated by activation of the *Tg*GRA2 promoter*,* resulting in its significant overexpression. In Western blot analysis of the lysates of RH*Δku80 Tguprt*-*Cpgp40* mutant, a ~ 50 kDa protein was recognized by HA monoclonal antibody, Strep monoclonal antibody, and GP40 monoclonal antibody, respectively (Fig. 2b). Therefore, the protein recognized was considered rec*Tg*GP40-Twinstrep-6 × HA. In addition, the size of rec*Tg*GP40-Twinstrep-6 × HA is comparable to that of the reported native GP40 with post-translational modification, indicating O-linked glycosylation of the recombinant protein. In the IFA analysis, *Cp*GP40 was co-localized with the Twin-Strep tag and HA tag, respectively, further supporting the fusion of *Cp*GP40 with the tags in *T. gondii* (Fig. 3a, b).

### Distribution of rec*Tg*GP40 and rec*Tg*GP15 in *T. gondii*

IFA analysis of the RH*Δku80* mutant (*Tguprt*-*Cpgp40* and *Tguprt*-*Cpgp15Δgpi*) showed that rec*Tg*GP40-Twinstrep-6 × HA was distributed in both the cytoplasm of *T. gondii* and parasitophorous vacuole (Fig. 3a, c), while rec*Tg*GP15-6 × HA without GPI was only distributed in the cytoplasm (Fig. 3a). Because rec*Tg*GP40 has a signal peptide, it can be secreted outside the tachyzoites and into the parasitophorous vacuole. However, rec*Tg*GP15 has neither signal peptide nor GPI anchor, so it can only be distributed in the cytoplasm. This suggested two potential approaches for subsequent purification of rec*Tg*GP40. Freshly egressed tachyzoites can be lysed and used for purification of recombinant proteins. Alternatively, cells can be lysed directly prior to parasite egress, and the lysate supernatant can be collected for purification of the recombinant proteins.

### Purification of rec*Tg*GP40

**Fig. 4:**
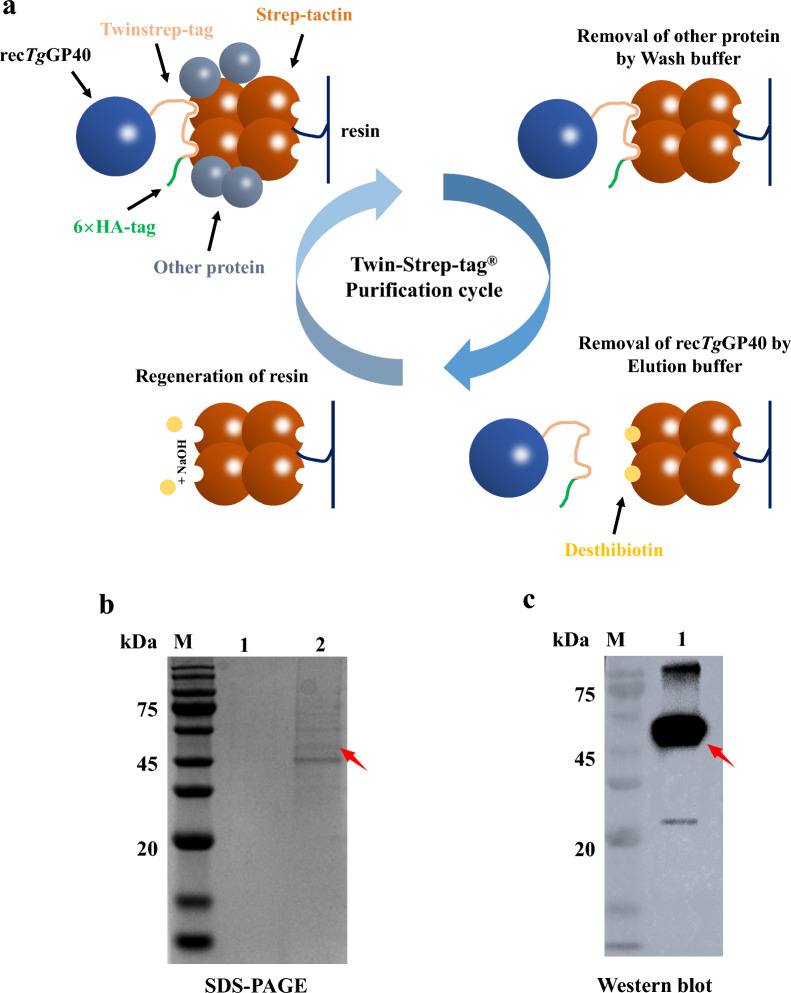
Production and purification of GP40 of *Cryptosporidium parvum* in *Toxoplasma gondii*. **a** Purification process of GP40 in *T. gondii* using Strep-TactinXT. The parasite lysis supernatant was coupled to a Strep-Tactin resin column. Due to the low affinity of Strep-Tactin for non-specific interactions, other proteins were readily washed away even under mild physiological conditions. The purified rec*Tg*GP40 was eluted by the introduction of low concentrations of desthiobiotin. This step, based on competitive displacement, enhances specificity while maintaining the overall buffer conditions (such as pH and ionic strength) unaltered. As a result, highly purified rec*Tg*GP40 was obtained and the function of the target protein was preserved; 10 mM NaOH solution was used to regenerate the Strep-Tactin column. **b** The purified rec*Tg*GP40 protein was visualized by Coomassie Brilliant Blue staining of SDS-PAGE gels (sodium dodecyl sulfate–polyacrylamide gel electrophoresis): Lane 1: purified rec*Tg*GP40 before concentration; lane 2: purified rec*Tg*GP40 after concentration. The red arrow indicates the rec*Tg*GP40 glycoprotein band. **c** The purified protein was analyzed by Western blotting with mAb HA. Lane 1: rec*Tg*GP40 after purification

IFA and Western blot analyses both indicated that rec*Tg*GP40-Twinstrep-6 × HA was abundantly expressed in the cytoplasm of the RH*Δku80 Tguprt*-*Cpgp40* mutant (Figs. 3a, 4b). Therefore, freshly egressed tachyzoites of the mutant were collected and subjected to lysis for protein purification. Rec*Tg*GP40-Twinstrep-6 × HA was purified using Strep-TactinXT through the Twin-Strep tag (Fig. 4a) and then concentrated by dialysis. In SDS-PAGE and Western blot analyses of the concentrated solution, several bands were present including one of ~ 50 kDa (Fig. 4b). In the corresponding Western blot analysis, a strong band of ~ 50 kDa was recognized by HA monoclonal antibody (Fig. 4c). Because the size of the band was comparable to the predicted size of rec*Tg*GP40, we supposed the recombinant protein is rec*Tg*GP40. In the mass spectrometry analysis of the band, seven peptide sequences yielded were compatible to the GP60 sequence of IIdA20G1-HLJ isolate (Protein ID: AZJ53484.1), which confirmed the *Cp*GP40 identity of the ~ 50 kDa band (Table 1). However, probably because of its low expression, rec*Tg*GP15 was not detectable in Western blot, and therefore could not be purified.

**Table 1:** Peptides of rec*Tg*GP40 matching *Cp*GP40 obtained by LC/MS

Start–end	Numbers	Sequence
70–121	1	TGEEVGNPGSEGQDGKGDTEETEDNQTESTVSQNTPAQTEGTTTETTEAAPK
122–142	3	KECGTSFVMWFGEGVPVASLK
143–162	2	CGDYTMVYAPEKDKTDPAPR
163–183	2	YISGEVTSVTFEKQESTVTIK
184–218	3	VNNVEFSTLSTSSSSPTENSGSAGQVPSEFGPASK
219–233	2	GSGSTQLWSHPQFEK
234–253	2	GGGSGGGSGGSAWSHPQFEK

## Discussion

GP60 is one of the most prominent mucin proteins in *Cryptosporidium* and is involved in the invasion of the parasites. Upon expression, it is cleaved into GP40 and GP15, with the former containing several O-linked glycosylation sites. In this study, we employed CRISPR/Cas9 technology to insert *GP40* and *GP15* into the *UPRT* locus of *T. gondii*. The *T. gondii* mutants generated can stably express GP40 and GP15. The recombinant GP15 without the GPI anchor localized in the cytoplasm of *T. gondii*. In contrast, the recombinant GP40 localized in the tachyzoite cytoplasm and parasitophorous vacuole and probably underwent O-linked glycosylation in *T. gondii* like that in *Cryptosporidium*. Using Strep-TactinXT, we recovered a large amount of rec*Tg*GP40, which should be useful for further investigation of *Cryptosporidium* mucins.

The mutants of *T. gondii* generated using CRISPR/Cas9 technology are capable of stable expression of *Cryptosporidium* proteins. *Toxoplasma gondii* can be easily cultured and genetically manipulated in vitro, making it a suitable choice for overexpressing proteins of *C. parvum*, which also belongs to Apicomplexa. Previously, *Cryptosporidium* GP60 has been expressed heterologously in *T. gondii* [16]. Subsequently, other *Cryptosporidium* mucins, P23 and Muc4, were expressed in *T. gondii* [27, 28]. However, in these studies, the heterogenous proteins were expressed through the plasmids transiently transfected into *T. gondii,* and the plasmids could be lost during the passages [29, 30]. In the current study, the heterologous genes of *Cryptosporidium* were integrated into the chromosome of *T. gondii* using CRISPR/Cas9 technology. GP40 was overexpressed in the RH*Δku80 Tguprt*-*Cpgp40* mutants as demonstrated by Western blot and IFA analyses. Therefore, this approach has an improved stability and reliability for the overexpression of target genes of *Cryptosporidium*.

The rec*Tg*GP40 is probably glycosylated in a manner analogous to native expression of the protein. Native *Cp*GP40 contains a signal peptide and several O-linked glycosylation sites [9, 12]. Signal peptides play crucial roles in directing proteins to the endoplasmic reticulum and Golgi apparatus for post-translational processing, including glycosylation [31, 32]. Therefore, the signal peptide of *Cp*GP40 was retained during the overexpression in *T. gondii* in this study. The similarity in the size of rec*Tg*GP40 and native *Cp*GP40 suggested the glycosylation of the recombinant protein in *T. gondii*. In addition, rec*Tg*GP40 was distributed in the cytoplasm of tachyzoites and the parasitophorous vacuoles, indicating secretion of the protein under the direction of the signal peptide. This is inconsistent with the surface localization of GP40 in *C. parvum* and transiently transfected *T. gondii* [16, 33]*.* The disruption of surface localization of rec*Tg*GP40 is probably due to the absence of GP15, which is associated with GP40 and attached to the membrane.

GP15 without a GPI anchor was only expressed in the cytoplasm. GPI is a common structural motif in apicomplexans, serving as anchors of some proteins for the attachment to cell membrane [34, 35]. In a previous study, GP15 was expressed in *T. gondii* with and without the GPI anchor [16]. The result showed that GP15 was unable to localize to the membrane of tachyzoites in absence of the GPI anchor. In IFA analysis, we observed rec*Tg*GP15 in the cytoplasm rather than on the surface of tachyzoites. Therefore, the localization of GP15 on the cell membrane is indeed GPI-dependent, at least within the heterologous expression system.

Combined with the Twin-Strep-based purification, the heterologous expression system established here allowed a high yield of purified rec*Tg*GP40 at a low cost. Because *C. parvum* is difficult to propagate in vitro and in vivo, native GP40 can only be isolated in limited amounts from purified *C. parvum* oocysts. The amount of recombinant GP40 expressed by transiently transfected *T*. *gondii* was limited, which was only detectable by silver staining [17]. Similarly, the heterologous expression of other *C. parvum* mucin proteins in *T. gondii*, such as P23 and Muc4, resulted in poor yields of recombinant proteins [27, 28]. These could be due to the loss of the plasmid containing the target genes and complicated purification procedure using a HA tag. In this study, stable heterologous expression of GP40 was achieved by inserting *GP40* into the chromosome of *T. gondii* using CRISPR/Cas9 technology. In addition, a Twin-Strep tag was fused to the C-terminal of GP40 for purification. Compared to the purification using a HA tag, the purification using the Twin-Strep tag was achieved by specific binding between the Strep-tag and the Strep-Tactin affinity chromatography resin, which was more efficient and cost effective [28, 36]. The high efficiency of the heterologous expression and Strep-based purification was supported by a visible band of ~ 50 kDa in SDS-PAGE (Fig. 4b), which was confirmed to be rec*Tg*GP40 by LC/MS analysis.

## Conclusions

In conclusion, we have established a heterologous expression system of *Cryptosporidium* mucin glycoproteins in *T. gondii* using CRISPR/Cas9 technology. The system allowed stable expression of GP40 and GP15 of *C. parvum* in *T. gondii*. In addition, we fused GP40 with a Twin-Strep-tag and HA tag, and a significant amount of rec*Tg*GP40 was purified using Strep-TactinXT resin at an affordable cost. Therefore, the novel approach and concept present here should be useful for the further study of *Cryptosporidium* mucins.

### Supplementary Information

**Additional file 1: Table S1.** Primers used in this study.

## Data Availability

All data are included as tables and figures within the article.

## Abbreviations

GP60: 60 KDa glycoprotein
GP40: 40 KDa glycoprotein
GP15: 15 KDa glycoprotein
GPI: Glycosyl phosphatidyl inositol
UPRT: Uracil phosphoribosyl transferase
CRISPR: Clustered regularly interspaced short palindromic repeats
Cas9: CRISPR-associated protein 9
PVDF: Polyvinylidene fluoride
TBST: Tris-buffered saline with Tween-20
IFA: Immunofluorescence assay
